# Pneumopéricarde compliquant un traumatisme thoraco-abdominal par coup de sabot

**DOI:** 10.11604/pamj.2014.19.132.5412

**Published:** 2014-10-07

**Authors:** Réda Guemmoune, Nabil Kanjaa

**Affiliations:** 1 Service de Réanimation Anesthésie Polyvalente, CHU Hassan II, Fès, Maroc

**Keywords:** Pneumopéricarde, traumatisme, thorax, Pneumopericarditis, traumatism, chest

## Image en medicine

Il n'existe que peu d’études consacrées aux accidents d’équitation. Avec une incidence de 36/100000 pratiquants, et un taux de mortalité de 0.06/100000, la prévalence dans la population générale reste faible, mais la gravité de ces accidents est liée à leur violence. Nous rapportons le cas d'un patient de 17 ans, victime d'un traumatisme thoraco-abdominal par coup de sabot. Reçu en état de choc aux urgences, avec une sensibilité abdominale, des sueurs profuses, des conjonctives décolorées, une tachycardie à 120bpm, et une tension artérielle à 80/50 mmHg. Le bilan lésionnel après stabilisation objectivait au niveau thoracique: un important emphysème sous cutané, un pneumothorax gauche, un pneumomediastin et une solution de continuité de la face antérieure du péricarde avec pneumopéricarde, et au niveau abdominal: un éclatement de la rate avec un hématome périsplénique et un épanchement intrapéritonéal de grande abondance. Le patient est admis au service de Réanimation après drainage thoracique et splénectomie d'hémostase réalisée en urgence. Le drainage pleural simple a permis le drainage péricardique.

**Figure 1:**
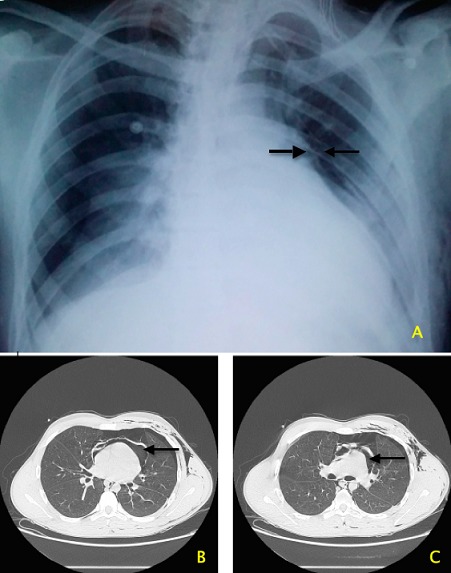
A) radio thoracique standard de face, mettant en évidence une hyperclarté aérique refoulant la ligne péricardique de tonalité tissulaire; B) scanner thoracique en coupe axiale, épanchement aérique intrapéricardique; C) scanner thoracique en coupe axiale, solution de continuité péricardique avec épanchement aérique intrapéricardique

